# Differential Neuroinflammatory Response in Male and Female Mice: A Role for BDNF

**DOI:** 10.3389/fnmol.2019.00166

**Published:** 2019-07-17

**Authors:** Andrea Carlo Rossetti, Maria Serena Paladini, Ada Trepci, Anne Mallien, Marco Andrea Riva, Peter Gass, Raffaella Molteni

**Affiliations:** ^1^Department of Medical Biotechnology and Translational Medicine, University of Milan, Milan, Italy; ^2^Department of Pharmacological and Biomolecular Sciences, University of Milan, Milan, Italy; ^3^Department of Psychiatry and Psychotherapy, Central Institute of Mental Health, Medical Faculty Mannheim, Heidelberg University, Heidelberg, Germany

**Keywords:** brain-derived neurotrophic factor, lipopolysaccharide, sex, neuroinflammation, hippocampus

## Abstract

A growing body of evidence supports the close relationship between major depressive disorder (MDD), a severe psychiatric disease more common among women than men, and alterations of the immune/inflammatory system. However, despite the large number of studies aimed at understanding the molecular bases of this association, a lack of information exists on the potential cross-talk between systems known to be involved in depression and components of the inflammatory response, especially with respect to sex differences. Brain-derived neurotrophic factor (BDNF) is a neurotrophin with a well-established role in MDD etiopathology: it is altered in depressed patients as well as in animal models of the disease and its changes are restored by antidepressant drugs. Interestingly, this neurotrophin is also involved in the inflammatory response. Indeed, it can be secreted by microglia, the primary innate immune cells in the central nervous system whose functions may be in turn regulated by BDNF. With these premises, in this study, we investigated the reciprocal impact of BDNF and the immune system by evaluating the neuroinflammatory response in male and female BDNF-heterozygous mutant mice acutely treated with the cytokine-inducer lipopolysaccharide (LPS). Specifically, we assessed the potential onset of an LPS-induced sickness behavior as well as changes of inflammatory mediators in the mouse hippocampus and frontal cortex, with respect to both genotype and sex. We found that the increased inflammatory response induced by LPS in the brain of male mice was independent of the genotype, whereas in the female, it was restricted to the heterozygous mice with no changes in the wild-type group, suggestive of a role for BDNF in the sex-dependent effect of the inflammatory challenge. Considering the involvement of both BDNF and neuroinflammation in several psychiatric diseases and the diverse incidence of such pathologies in males and females, a deeper investigation of the mechanisms underlying their interaction may have a critical translational relevance.

## Introduction

Major depressive disorder (MDD) is a severe psychiatric disease affecting almost 10% of the general population and estimated to become the second leading cause of disability by 2020 (Bromet et al., 2011). In addition to the symptom heterogeneity among patients and to its complex etiology (Otte et al., 2016), MDD presents a sexually dimorphic nature, characterized by a twofold greater risk in females to develop the pathology (Brody et al., 2018; LeGates et al., 2019). On this basis and given the different immunological response in terms of innate and adaptive systems among sexes (Klein and Flanagan, 2016), it is plausible that sex-based differences in immune response might play an important role to the diverse vulnerability/incidence to psychiatric disorders (Derry et al., 2015; Rubinow and Schmidt, 2019). In the last years, growing evidence suggests that alterations in the inflammatory/immune response may contribute to the susceptibility for different psychiatric conditions, including MDD (Müller, 2014). Particularly, the increased levels of peripheral and central inflammatory markers observed in a large subset of MDD subjects (Raison et al., 2006; Howren et al., 2009; Dowlati et al., 2010), the high co-morbidity between MDD and various non-psychiatric illnesses associated with inflammatory conditions (Anisman, 2008; Berge and Riise, 2015; Réus et al., 2017), and the development of MDD in a high percentage of patients under interferon regimen (Raison et al., 2009; Udina et al., 2012) clearly support the relevance of a neuroinflammatory component in MDD. In addition, while changes in immune/inflammatory response have been found in MDD experimental models based on both the genetic and the environmental components of the disease (Chourbaji et al., 2011; Couch et al., 2013; Macchi et al., 2013; Rossetti et al., 2016; Wang et al., 2018), several studies indicate the immune/inflammatory system as a potential target for the pharmacological treatment of MDD (Molteni et al., 2013; Kv et al., 2018; Rossetti et al., 2018a,b; Lu et al., 2019).

It is known that this complex psychopathology affects multiple molecular systems such as neurotransmitters, hormones, and mediators of neuronal plasticity. Among those, the neurotrophin brain-derived neurotrophic factor (BDNF) plays a crucial role. Indeed, BDNF levels are reduced in depressed subjects and the neurotrophin represents a key step in long-term adaptive changes brought about by antidepressant drugs (Berton and Nestler, 2006; Martinowich et al., 2007; Molendijk et al., 2014; Castrén and Kojima, 2017). In addition, BDNF is dramatically affected by inflammatory insults (Calabrese et al., 2014; Lima Giacobbo et al., 2018): the administration of lipopolysaccharide (LPS) or pro-inflammatory cytokines in rodents has been shown to decrease its levels in the cortex and hippocampus (Guan and Fang, 2006; Schnydrig et al., 2007; Patterson, 2015), while the therapeutic treatment with interferons alters BDNF expression in human subjects (Capuron et al., 2004; Kenis et al., 2011; Lotrich et al., 2013). Moreover, microglial cells, the macrophage resident population within the brain and one of the crucial components of the neuroinflammatory response, express BDNF mRNA, secrete the neurotrophin following stimulation, and are regulated by BDNF signaling (Nakajima et al., 2001; Ferrini and De Koninck, 2013).

On this basis, the main goal of our study was to investigate the potential link between neuroinflammation and BDNF in male and female mice by evaluating if and how the inflammatory response might be altered in conditions characterized by compromised expression of the neurotrophin. Accordingly, we assessed the brain inflammatory response of male and female BDNF^+/–^ mice, which exhibit an approximately 50% reduction of BDNF protein and mRNA (Korte et al., 1995; Lyons et al., 1999; Sairanen et al., 2005; Chourbaji et al., 2012), after an acute systemic injection of LPS in comparison with wild-type mice. This experimental setting might actually provide important information regarding the sex-dependent pathological consequences of a dysregulated immune/inflammatory response, pointing out the role of BDNF. In line with our approach, it has been reported that some biological functions of the neurotrophin are different in male and female. For instance, the administration of BDNF may alleviate the pain induced by acetic acid in male rats but has no effect in females (Li et al., 2010). Moreover, different basal protein levels of the neurotrophin have been detected in humans (Hayley et al., 2015) and in rodents (Szapacs et al., 2004; Franklin and Perrot-Sinal, 2006) as well as a different activation of the BDNF signaling (Hill and van den Buuse, 2011).

Based on these considerations, we hypothesized that the altered interaction between the inflammatory system and BDNF may represent a potential candidate contributing to the sex-dependent differences in the inflammatory response that, in turn, might be associated with several psychiatric disorders such as MDD.

## Materials and Methods

### Animals

Wild-type and *BDNF*^+/–^ male and female littermate mice on a C57BL/6N background were bred as described earlier (Chourbaji et al., 2004). All the animals were housed individually at the age of 13–19 weeks in standard macrolon cages (type II—26 cm × 20 cm × 14 cm) with bedding and nesting material (paper tissue). They were acclimatized at least for 2 weeks to a reserved 12-h dark–light cycle (lights off 8 am to 8 pm) at 22 ± 1°C room temperature and the humidity was 35% as described earlier (Chourbaji et al., 2005). Animals received a standard pellet diet and water *ad libitum*. Lastly, to reduce the number of experimental biases, all the experiments were performed using blind numbers. All animal experiments were approved by the Animal Welfare Office of the Regierungspräsidium Karlsruhe, Germany.

### LPS Treatment and Behavioral Evaluation

After the acclimatization phase, wild-type and heterozygous mice were randomly divided to receive saline or LPS (LPS from *Escherichia coli*; serotype 026:B6; Sigma Aldrich). The bacterial toxin was dissolved in sterile, endotoxin-free isotonic saline and injected i.p. from 1 mg/ml stock solution. We decided to use a dosage of 400 μg/kg as a low dose of LPS able to induce only subtle changes in body weight and locomotor activity few hours from the administration, which disappeared within 24 h (Couch et al., 2016). In a pilot study, we compared this dosage with 830 μg/kg, which is similar to the amount used in several studies, finding that the two doses had the same impact on the body weight and locomotion (data not shown). The lowest dose of LPS was chosen as it is less aversive to the subjects and in order to avoid a massive activation of the inflammatory response that could have masked the molecular impact of the genotype and/or the sex of the animals. With this experimental design, we obtained eight groups of animals: wild-type mice treated with saline (male, *n* = 5; female, *n* = 6) or LPS (male, *n* = 6; female, *n* = 6); *BDNF*^+/–^ mice that received saline (male, *n* = 6; female, *n* = 6) or the bacterial toxin (male, *n* = 6; female, *n* = 6).

We assessed body weight before the beginning of the experiment (baseline), 1 h before LPS administration, and 6 and 24 h after the immune challenge. Moreover, 6 h after the injection, animals were tested with the Open Field (OF) test to evaluate alterations in the locomotor activity as previously reported (Zueger et al., 2005; Richter et al., 2011). Briefly, after a period of acclimatization to the room (30 min), mice were individually placed in a square-shaped, white, illuminated (25 lx) arena, measuring 50 × 50 cm^2^. The test, conducted in the active phase of the animals, was monitored from above for 10 min and recorded by a video camera (Sony CCD IRIS). For each subject, total distance moved and velocity were analyzed by a blinded experimenter using the image processing system Etho Vision 3.0 (Noldus Information Technology). Animals were sacrificed 24 h after LPS injection, the brains were harvested, and the hippocampus and frontal cortex were dissected on ice from both the hemispheres. The tissues were rapidly frozen on dry ice and stored at −80°C until the molecular analyses.

### RNA Preparation and Gene Expression Analyses

For gene expression analyses, total RNA was isolated from the different brain regions by single-step guanidinium isothiocyanate/phenol extraction using PureZol RNA isolation reagent (Bio-Rad Laboratories S.r.l.) according to the manufacturer’s instructions and quantified by spectrophotometric analysis. The samples were then processed for real-time polymerase chain reaction (PCR) as previously reported (Rossetti et al., 2016) to assess mRNA levels of target genes.

Briefly, an aliquot of each sample was treated with DNase to avoid DNA contamination and subsequently analyzed by TaqMan qRT-PCR instrument (CFX384 real-time system, Bio-Rad Laboratories S.r.l.) using the iScript one-step RT-PCR kit for probes (Bio-Rad Laboratories S.r.l). Samples were run in 384-well format in triplicate as multiplexed reactions with a normalizing internal control (β-*Actin*). Thermal cycling was initiated with incubation at 50°C for 10 min (RNA retrotranscription), and then at 95°C for 5 min (TaqMan polymerase activation). After this initial step, 39 cycles of PCR were performed. Each PCR cycle consisted of heating the samples at 95°C for 10 s to enable the melting process and then for 30 s at 60°C for the annealing and extension reactions. A comparative cycle threshold (Ct) method was used to calculate the relative target gene expression. Probe and primer sequences used were purchased from Applied Biosystem Italia and Eurofins MWG-Operon. A complete list of primers and probes is presented in Table 1.

**TABLE 1:** Sequences of forward and reverse primers and probes used in qRT-PCR analyses.

**Gene**	**Forward primer**	**Reverse primer**	**Probe**
Cd11b^*^	CATCCCATGACCTTCCAAGAG	GTGCTGTAGTCACACTGGTAG	CCACACTCTGTCCAAAGCCTTTTGC
Cx3cl1^*^	TCTTCCATTTGTGTACTCTGCT	GGACTCCTGGTTTAGCTGATAG	TGTCGCACATGATTTCGCATTTCGTC
Cx3cr1^*^	GTTATTTGGGCGACATTGTGG	ATGTCAGTGATGCTCTTGGG	TCTGGTGGGAAATCTGGTTGGTGGTC
β-Actin^*^	ACCTTCTACAATGAGCTGCG	CTGGATGGCTACGTACATGG	TCTGGGTCATCTTTTCACGGTTGGC
*Il-1β^∗∗^*	Mm00434228_m1		
*Il-6^∗∗^*	Mm00446190_m1		
*Tnf-*α^∗∗^	Mm00443258_m1		

Purchased from Eurofins MWG Operon (Germany)^*^ and Applied Biosystem (Italy)^∗∗^.

### Protein Extraction and Western Blot Analysis

Protein extracts were obtained as previously described (Rossetti et al., 2018a,b). Briefly, brain samples were manually homogenized using a glass–glass potter in a pH 7.4 cold isotonic buffer and then sonicated for 10 s at a maximum power of 10–15% (Bandelin Sonoplus). The homogenate was clarified by centrifugation to obtain a pellet (P1) enriched in nuclear components, which was resuspended in a hypotonic buffer. The supernatant (S1) was then centrifuged (13,000 × *g*; 15 min) to obtain a clarified fraction of cytosolic proteins (S2). The pellet (P2), corresponding to the crude membrane fraction, was resuspended in the same buffer used for the nuclear fraction. Total protein content was measured according to the Bradford Protein Assay procedure (Bio-Rad Laboratories), using bovine serum albumin as calibration standard. For the protein analysis, 10 μg of P2 protein lysates were run under reducing conditions on polyacrylamide gels and then transferred onto nitrocellulose membranes. Unspecific binding sites were blocked with 10% non-fat dry milk and then the membranes were incubated overnight with the primary antibody (TLR-4 Santa Cruz cat. sc-10741; 1:1,000), followed by a 1-h incubation at room temperature with a peroxidase-conjugated anti-rabbit IgG. Immunocomplexes were visualized by chemiluminescence using the ETA C2.0 (Cyanagen). Protein levels were calculated by measuring the optical density of the immunocomplexes using chemiluminescence (Chemidoc MP Imaging System, Bio-Rad Laboratories) and results were standardized on β-Actin (Sigma cat. A5441; 1:10000) bands at 43 kDa as internal control.

### Statistical Analyses

Behavioral data were analyzed using repeated measurement ANOVA (Time^*^LPS^*^Genotype): one-way ANOVA (Treatment) or two-way ANOVA (LPS^*^Genotype). When appropriate, Bonferroni *post hoc* tests were used to evaluate furtherdifferences between groups.

Molecular data were analyzed by two-way ANOVA, with treatment (Saline vs. LPS) or genotype (wild type vs. *BDNF*^+/–^) as independent factors. When appropriate, direct contrasts were analyzed with Fisher’s protected least significant difference (PLSD). All the molecular analyses were carried out in individual animals (independent determinations), and for graphic clarity, data are presented as mean percent ± standard error (SEM) of control group, with significance threshold set at *P* < 0.05.

## Results

### LPS Administration Alters the Body Weight and the Locomotion of Both Male and Female Animals

First, we have assessed the ability of LPS to induce the so-called sickness behavior by measuring the body weight of the animals and monitoring their locomotor activity in the OF test 6 h after the inflammatory challenge. Although it is known from the literature that at this time point LPS may cause behavioral consequences, our aim was to evaluate the influence of sex or genotype.

**FIGURE 1:**
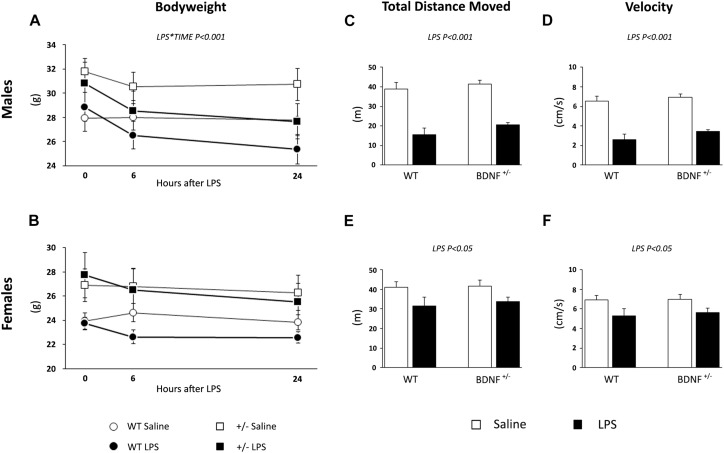
Evaluation of LPS-induced sickness behavior through body weight and locomotor activity. The body weight of both male **(A)** and female **(B)** animals was measured before LPS injection, 6 and 24 h after the immune challenge. After 6 h from the injection, we tested saline and LPS-treated animals performance with the OF test to assess the total distance traveled (males, **C**; females, **E**) and the velocity (males, **D**; females, **F**) as parameters of locomotion.

At baseline, wild-type and heterozygous mice showed differences in body weight that were statistically significant only in female animals as a result of the BDNF partial deletion (Genotype: *F*_1,20_ = 6.940, *P* < 0.05; Table 2 and Figures 1A,B). After LPS administration, we observed a decrease of body weight over time in male (LPS^*^Time, *F*_1,19_ = 17.046, *P* < 0.001; Figure 1A), while in female mice, the impact of the immune challenge was blunted by the basal genotype effect (Figure 1B).

**TABLE 2:** Baseline body weight of the animals used in this study.

	**Wild type**	***BDNF*^+/–^**
Males	28.48 ± 3.26	30.02 ± 3.73
Females	23.34 ± 1.95	25.90 ± 3.45

*The body weight was assessed before the beginning of the study. The data are presented as average weight ± standard deviation of 12 animals/group.*

In the OF test, we analyzed the total distance moved and the velocity of the animals 6 h after the exposure to the bacterial toxin. As shown in Figure 1C, the total distance moved by male mice treated with LPS was reduced when compared to the locomotion of saline-treated mice (LPS, *F*_1,23_ = 74.485, *P* < 0.001; Figure 1C) irrespective of the genotype. A similar, but milder effect was observed for female animals; indeed, LPS treatment significantly affected the distance traveled (*F*_1,20_ = 7.566, *P* < 0.05; Figure 1E) in both wild-type and heterozygous mice.

LPS also modulated the velocity of the animals during the locomotion test, which was reduced in both male (*F*_1,19_ = 73.859, *P* < 0.001; Figure 1D) and female mice (*F*_1,20_ = 7,582, *P* < 0.05; Figure 1F) without any effect of the genotype. Similar to what was previously observed, the magnitude of the LPS-induced changes was higher in male animals.

### Wild-Type and Heterozygous Mice Have the Same Basal Levels of TLR-4, Without Gender Differences

To exclude the idea that LPS could have acted on a different receptor background, we examined the protein levels of toll-like receptor 4 (TLR-4), the endogenous receptor for the bacterial toxin.

**FIGURE 2:**
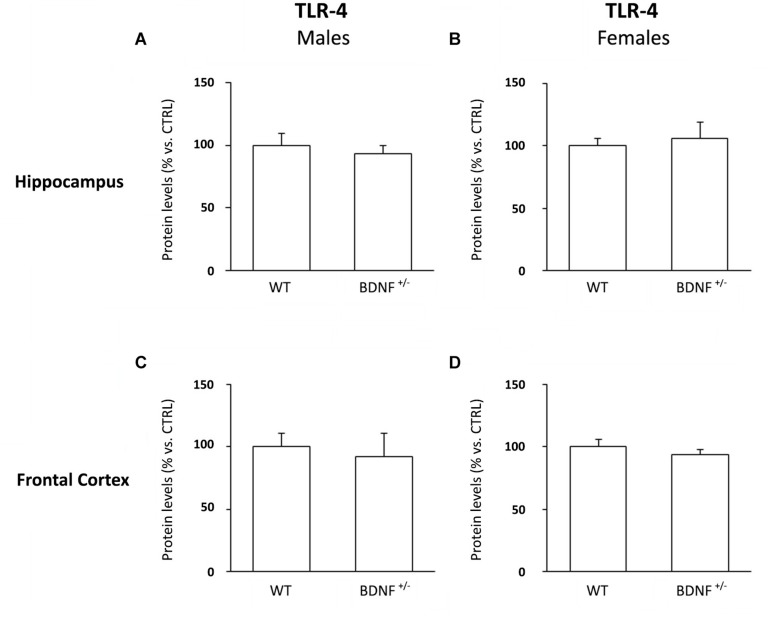
Western Blot Analysis of TLR-4 Basal Levels in the Hippocampus and in the Frontal Cortex. The protein levels of the toll like-4 receptor (TLR-4) were measured in the hippocampus and in the frontal cortex of male **(A,C)** and female **(B,D)** wild-type (WT) and BDNF heterozygous (BDNF^+/–^) mice treated with saline. The data, expressed as a percentage of the saline-injected wild-type mice (CTRL, set at 100%), represent the mean ± SEM of at least five independent determinations.

As shown in Figure 2, we did not find any difference among the experimental groups.

### The Immune Challenge Differentially Affects the Cytokine Expression Profile in the Hippocampus and Frontal Cortex of Male and Female Heterozygous Mice

Next, we investigated if sex or genotype may also influence also the molecular impact of the inflammatory challenge. With this aim, we analyzed the gene expression of the pro-inflammatory cytokines IL-1β, TNF-α, and IL-6 in the hippocampus and in the frontal cortex of wild-type and mutant mice of both sexes. The molecular analyses were performed 24 h after the immune challenge to avoid the peak of cytokine expression usually observed in the first hours from LPS injection, which could confound the influence of the other two variables.

**FIGURE 3:**
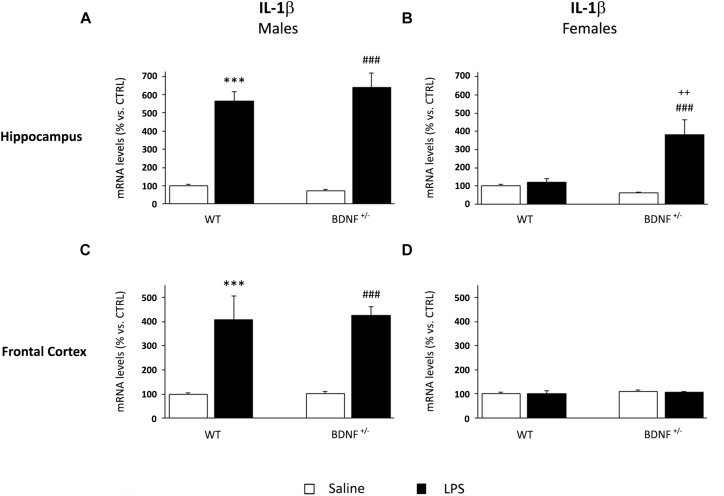
Gene expression analysis of IL-1β in the hippocampus and in the frontal cortex. The mRNA levels of the pro-inflammatory cytokine IL-1β were measured in the hippocampus and in the frontal cortex of male **(A,C)** and female **(B,D)** wild-type (WT) and BDNF heterozygous (BDNF^+/–^) mice 24 h after a single injection of lipopolysaccharide (LPS, 400 μg/kg i.p). in comparison with mice treated with saline. The data, expressed as a percentage of the saline-injected wild-type mice (CTRL, set at 100%), represent the mean ± SEM of at least six independent determinations. ^∗∗∗^*P* < 0.001 vs. CTRL; ^###^*P* < 0.001 vs. BDNF^+/–^; ^++^*P* < 0.01 vs. WT/LPS (Two-way ANOVA with PLSD).

IL-1β gene expression was significantly modulated by LPS administration in the hippocampus of both wild-type and heterozygous male mice (*F*_1,19_ = 179.8, *P* < 0.001). Specifically, the challenge markedly increased the pro-inflammatory cytokine without differences between the two genotypes (WT/LPS +465% vs. WT/SAL, *P* < 0.001; *BDNF*^+/–^/LPS +571% vs. *BDNF*^+/–^/SAL, *P* < 0.001; Figure 3A). Conversely, a different profile was observed in the hippocampus of female mice where the significant effect of the LPS injection (*F*_1,18_ = 14.17, *P* < 0.01) was restricted to the mutant animals, as indicated by the significant treatment^*^genotype interaction (*F*_1,18_ = 10.85, *P* < 0.01). Indeed, as shown in Figure 3, IL-1β mRNA levels were significantly induced by LPS only in the heterozygous mice (*BDNF*^+/–^/LPS +324% vs. *BDNF*^+/–^/SAL, *P* < 0.001; +262% vs. WT/LPS, *P* < 0.01; Figure 3B) with no changes in wild-type animals. Moreover, it has to be noted that the magnitude of the cytokine induction in female mice was lower with respect to male animals, although its basal expression was similar.

In the frontal cortex of male mice, IL-1β expression was similar to that observed in the hippocampus. LPS administration, indeed, significantly increased the mRNA of the pro-inflammatory cytokine (*F*_1,22_ = 45.49, *P* < 0.001) in both wild-type (WT/LPS +309% vs. WT/SAL, *P* < 0.001) and heterozygous male mice (*BDNF*^+/–^/LPS +324% vs. *BDNF*^+/–^/SAL, *P* < 0.001; Figure 3C) without differences between the two experimental groups. Conversely, no changes in IL-1β levels were found in the frontal cortex of female mice exposed to LPS (Figure 3D).

**FIGURE 4:**
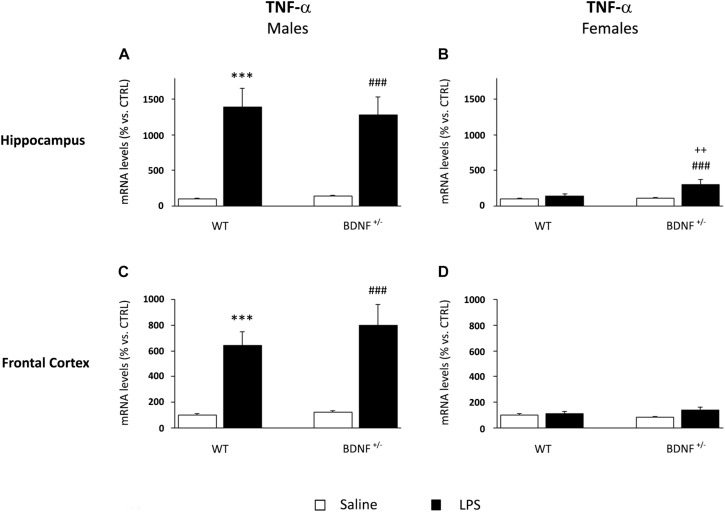
Gene expression analysis of TNF-α in the hippocampus and in the frontal cortex. The mRNA levels of the pro-inflammatory cytokine TNF-α were measured in the hippocampus and in the frontal cortex of male **(A,C)** and female **(B,D)** wild-type (WT) and BDNF heterozygous (BDNF^+/–^) mice 24 h after a single injection of lipopolysaccharide (LPS, 400 μg/kg i.p). in comparison with mice treated with saline. The data, expressed as a percentage of the saline-injected wild-type mice (CTRL, set at 100%), represent the mean ± SEM of at least six independent determinations. ^∗∗∗^*P* < 0.001 vs. CTRL; ^###^*P* < 0.01 vs. BDNF^+/–^; ^++^*P* < 0.01 vs. WT/LPS (Two-way ANOVA with PLSD).

Similarly to what was observed for IL-1β, the mRNA levels of TNF-α were significantly up-regulated by the LPS treatment in male mice (*F*_1,20_ = 42.58, *P* < 0.001), an effect independent of the genotype. In fact, the inflammatory challenge strongly induced the expression of TNF-α in the hippocampus in both wild-type (WT/LPS +1287% vs. WT/SAL, *P* < 0.001; Figure 4A) and mutant mice (*BDNF*^+/–^/LPS +1142% vs. *BDNF*^+/–^/SAL, *P* < 0.001; Figure 4A) without any significant difference between the genotypes. On the contrary, in female mice, the increased TNF-α gene expression by LPS was limited to the mutant animals, as indicated by the significant effects of LPS (*F*_1,18_ = 6.74, *P* < 0.05), genotype (*F*_1,18_ = 10.7, *P* < 0.01), and interaction between LPS^*^Genotype (*F*_1,18_ = 4.95, *P* < 0.05). Again, this increase was less pronounced with respect to the modulations observed in male mice (*BDNF*^+/–^/LPS +186% vs. *BDNF*^+/–^/SAL, *P* < 0.01; +164% vs. WT/LPS ^∗∗^*P* < 0.01; Figure 4B).

In the frontal cortex, the gene expression profile of TNF-α was qualitatively comparable to that observed in the hippocampus, although the effect of the inflammatory challenge was lower. As shown in Figure 4C, we found a significant increase of TNF-α mRNA levels after LPS injection (*F*_1,22_ = 42.43, *P* < 0.001) in both wild-type (WT/LPS +544% vs. WT/SAL, *P* < 0.001) and mutant male mice (*BDNF*^+/–^/LPS +678% vs. *BDNF*^+/–^/SAL, *P* < 0.001). Interestingly, a slight but significant modulation of TNF-α by LPS (*F*_1,21_ = 4.45, *P =* 0.05) was also observed in heterozygous female mice (*BDNF*^+/–^/LPS +55% vs. *BDNF*^+/–^/SAL, *P* < 0.01; Figure 4D).

**FIGURE 5:**
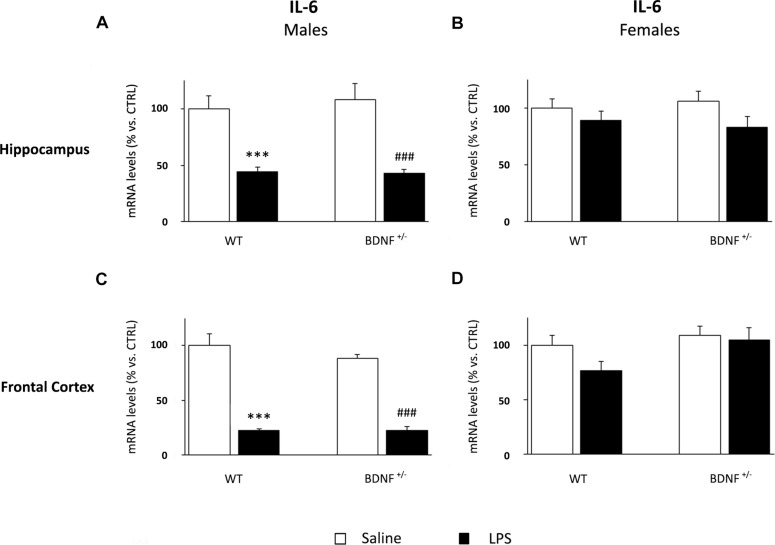
Gene expression analysis of IL-6 in the hippocampus and in the frontal cortex. The mRNA levels of the pro-inflammatory cytokine IL-6 were measured in the hippocampus and in the frontal cortex of male **(A,C)** and female **(B,D)** wild-type (WT) and BDNF heterozygous (BDNF^+/–^) mice 24 h after a single injection of lipopolysaccharide (LPS, 400 μg/kg i.p). in comparison with mice treated with saline. The data, expressed as a percentage of the saline-injected wild-type mice (CTRL, set at 100%), represent the mean ± SEM of at least six independent determinations. ^∗∗∗^*P* < 0.001 vs. CTRL; ^###^*P* < 0.001 vs. BDNF^+/–^ (Two-way ANOVA with PLSD).

The expression of IL-6 was differentially modulated by LPS administration if compared to the other cytokines. Indeed, the inflammatory challenge significantly decreased IL-6 mRNA levels (*F*_1,19_ = 42.84, *P* < 0.001) in both wild-type (WT/LPS -56% vs. WT/SAL, *P* < 0.001) and heterozygous mice (*BDNF*^+/–^/LPS -65% vs. *BDNF*^+/–^/SAL, *P* < 0.001; Figure 5A). On the contrary, we did not observe any significant change in female mice (Figure 5B).

In line with the observed expression profile in the hippocampus, the expression of IL-6 was significantly down-regulated by LPS also in the frontal cortex of male mice (*F*_1,19_ = 123.9, *P* < 0.001). The impact of the immune challenge altered the expression of the cytokine in wild-type (WT/LPS -77% vs. WT/SAL, *P* < 0.001) as well as mutant animals (*BDNF*^+/–^/LPS -66% vs. *BDNF*^+/–^/SAL, *P* < 0.001; Figure 5C), an effect even greater in this brain region, when compared to the hippocampus. IL-6 expression was not affected in the frontal cortex of female mice (Figure 5D).

### LPS Administration Differentially Modulates Microglial Markers in Male and Female Brain, With a Specific Influence of the Genotype

Considering the pivotal role of microglia in the immune response within the central nervous system, we analyzed the expression of a marker of microglia activation, namely, CD11b, and two molecules involved in the control of microglia response, such as fractalkine (CX3CL1) and its receptor (CX3CR1).

**FIGURE 6:**
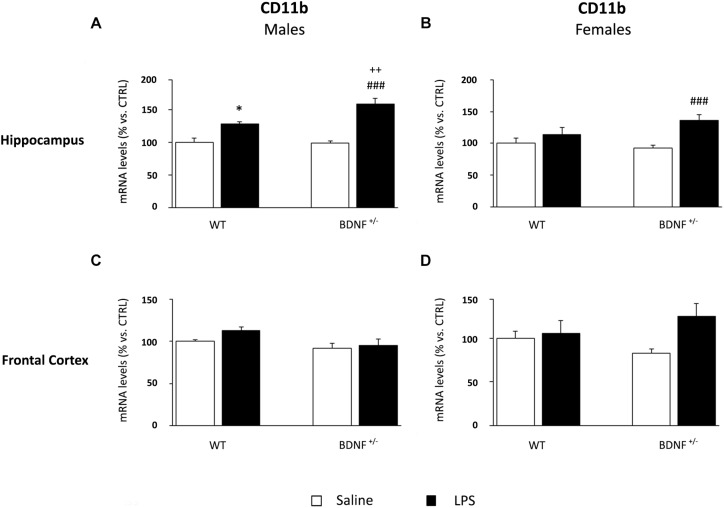
Gene expression analysis of CD11b in the hippocampus and in the frontal cortex. The mRNA levels of the microglial marker CD11b were measured in the hippocampus and in the frontal cortex of male **(A,C)** and female **(B,D)** wild-type (WT) and BDNF heterozygous (BDNF^+/–^) mice 24 h after a single injection of lipopolysaccharide (LPS, 400 μg/kg i.p). in comparison with mice treated with saline. The data, expressed as a percentage of the saline-injected wild-type mice (CTRL, set at 100%), represent the mean ± SEM of at least six independent determinations. ^*^P < 0.05 vs. CTRL; ^###^*P* < 0.001 vs. BDNF+/−/LPS; ^++^*P* < 0.01 vs. WT/LPS (two-way ANOVA with PLSD).

The analyses on the hippocampus of male mice indicated that the expression of CD11b was significantly modulated by the inflammatory challenge (*F*_1,23_ = 48.93, *P* < 0.001) and by the genotype (*F*_1,23_ = 5.05, *P* < 0.05). As shown in Figure 6A, CD11b expression was increased in both wild-type (WT/LPS +27% vs. WT/SAL, *P* < 0.01) and *BDNF* heterozygous mice (*BDNF*^+/–^/LPS +60% vs. *BDNF*^+/–^/SAL, *P* < 0.001). Interestingly, this effect was significantly higher in the mutant animals (*BDNF*^+/–^/LPS +31% vs. WT/LPS, *P* < 0.01) as indicated by the LPS^*^Genotype interaction (*F*_1,23_ = 6.85, *P* < 0.05). In the hippocampus of female mice, the expression of the microglial marker was significantly affected by the immune challenge (*F*_1,23_ = 13.06, *P* < 0.01) only in mutant animals (*BDNF*^+/–^/LPS +44% vs. *BDNF*^+/–^/SAL, *P* < 0.001; Figure 6B). Conversely, in the frontal cortex, we did not observe any significant modulation of the expression of CD11b by LPS or by the genotype, neither in male nor in female mice (Figures 6C,D).

**FIGURE 7:**
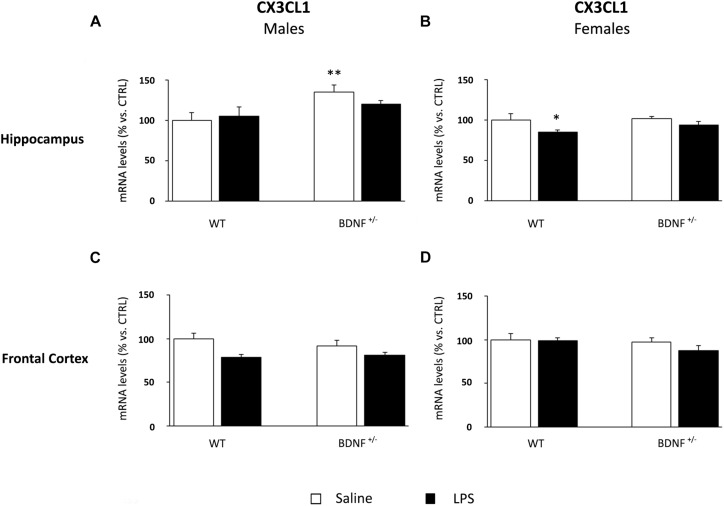
Gene expression analysis of CX3CL1 in the hippocampus and in the frontal cortex. The mRNA levels of CX3CL1 were measured in the hippocampus and in the frontal cortex of male **(A,C)** and female **(B,D)** wild-type (WT) and BDNF heterozygous (BDNF^+/–^) mice 24 h after a single injection of lipopolysaccharide (LPS, 400 μg/kg i.p). in comparison with mice treated with saline. The data, expressed as a percentage of the saline-injected wild-type mice (CTRL, set at 100%), represent the mean ± SEM of at least six independent determinations. ^*^P < 0.05 and ^∗∗^*P* < 0.01 vs. CTRL (two-way ANOVA with PLSD).

The gene expression analyses of fractalkine in the hippocampus of male mice indicated a significant impact of the genotype (*F*_1,22_ = 8.72, *P* < 0.01). Indeed, the basal levels of CX3CL1 were significantly higher only in BDNF heterozygous mice if compared to control mice (*BDNF*^+/–^/SAL +36% vs. WT/SAL, *P* < 0.01; Figure 7A). Conversely, the mRNA levels of CX3CL1 in female mice were modulated by LPS administration (*F*_1,23_ = 5.07, *P* < 0.05) only in wild-type animals (WT/LPS -16% vs. WT/SAL, *P* < 0.05; Figure 7B). No significant changes were found in the prefrontal cortex (Figures 7C,D).

**FIGURE 8:**
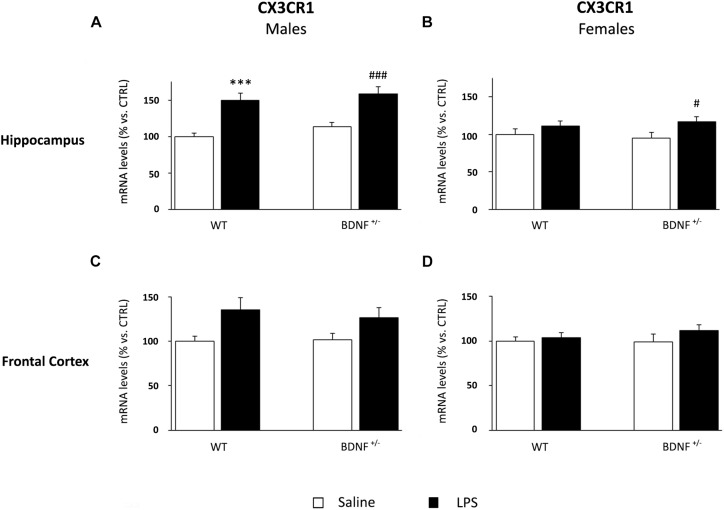
Gene expression analysis of CX3CR1 in the hippocampus and in the frontal cortex. The mRNA levels of CX3CR1 were measured in the hippocampus and in the frontal cortex of male **(A,C)** and female **(B,D)** wild-type (WT) and BDNF heterozygous (BDNF^+/–^) mice 24 h after a single injection of lipopolysaccharide (LPS, 400 μg/kg i.p). in comparison with mice treated with saline. The data, expressed as a percentage of the saline-injected wild-type mice (CTRL, set at 100%), represent the mean ± SEM of at least six independent determinations. ^∗∗∗^*P* < 0.001 vs. CTRL; ^#^*P* < 0.05 and ^###^*P* < 0.001 vs. BDNF^+/–^ mice (two-way ANOVA with PLSD).

Despite the slight modulation of fractalkine observed in the hippocampus of male mice, the inflammatory challenge had significant effect on CX3CR1 (*F*_1,22_ = 34.17, *P* < 0.001). Specifically, the mRNA levels of the receptor were significantly increased by LPS in both wild-type (WT/LPS +50% vs. WT/SAL, *P* < 0.001; Figure 8A) and BDNF heterozygous mice (*BDNF*^+/–^/LPS +45% vs. *BDNF*^+/–^/SAL, *P* < 0.001; Figure 8A). In female mice, the hippocampal CX3CR1 expression was up-regulated following the treatment (*F*_1,24_ = 5.54, *P* < 0.05), but only in heterozygous mice (*BDNF*^+/–^/LPS +22% vs. *BDNF*^+/–^/SAL, *P* < 0.05; Figure 8B).

Although we observed a similar modulation also in the frontal cortex of male (Figure 8C) and female mice (Figure 8D), neither the impact of the immune challenge nor the *BDNF* mutation significantly affected the expression of fractalkine receptor.

## Discussion

In this study, we show that the partial deletion of BDNF influences the inflammatory response to LPS in a sex-specific manner. Specifically, we found that the molecular impact of the inflammatory challenge was different in the two sexes: high in male mice independently from the genotype, almost null in wild-type female mice, and mild in the BDNF heterozygous female mice. This effect was mainly observed in the hippocampus, suggesting a pivotal role for the neurotrophin in the inflammatory response of this area of the female brain.

The systemic injection of LPS in the rodent mimics a gram-negative bacterial infection that induces a massive inflammatory response with a release of pro-inflammatory cytokines and the insurgence of the so-called “sickness behavior,” characterized by decreased motor activity, social withdrawal, reduced food and water intake, and altered cognition within a few hours from the LPS administration (Dantzer et al., 2008). Likewise, we found reduced body weight and decreased locomotor activity 6 h after the LPS administration, an effect independent from genotype but influenced by sex. It is important to mention that animals of both sexes were single caged. While single housing could have been more favorable for male mice due to less social distress and aggressive behaviors (Chourbaji et al., 2005; Kamakura et al., 2016; Kappel et al., 2017), individual housing could have affected the behavior of female mice. Nevertheless, the baseline levels of the parameters analyzed in the OF were comparably similar between sexes, suggesting that the housing condition apparently did not alter the behavioral outcomes analyzed in our study. Male mice showed a stronger induction of sickness behavior if compared to the female counterpart, as indicated by a greater weight loss and a worse OF performance, in line with previous published data (Cai et al., 2016). Similarly, the neuroinflammatory response was stronger in males than in females when assessed at molecular levels. Thus, the mRNA levels of IL-1β and TNF-α were increased in the hippocampus and frontal cortex of male mice but not in females. These changes were paralleled by a strong decrease of IL-6 transcripts in male mice 24 h after LPS, an effect that was already reported in other studies (Schneiders et al., 2015) and could probably be ascribed to the inhibitory activity of the suppressor of cytokine signaling 3 (SOCS3), a protein controlling the negative feedback regulation of IL-6 (Babon et al., 2014). Since it has been shown that TNF-α is able to induce SOCS3 protein levels, thus modulating IL-6 pathway (Dagvadorj et al., 2010), the cross-talk between these two cytokines might explain the decrease of IL-6 observed only in the brain of male mice where the levels of TNF-α were dramatically increased.

It has to be noted that, despite the observed sex-dependent differences in LPS response, the toxin is acting on the same receptor background, as no basal changes in the expression of TLR-4, the endogenous receptor for LPS, were observed in any experimental group.

The differences in the inflammatory response among the sexes are actually well known (Klein and Flanagan, 2016); however, beside a role for genetic and/or hormonal mediators, a lack of information exists regarding the underlying mechanisms.

Among the molecular systems that might contribute to such differential response, our data suggest a role for the neurotrophin BDNF. Indeed, the *BDNF*^+/–^ female mice showed a significant LPS-dependent increase of the abovementioned cytokines, mainly in the hippocampus, an effect that—to our knowledge—has never been reported. To further analyze the different expression profile between male and female heterozygous mice, we focused our attention on microglia, the immune resident cell population of the brain. In addition to their role as first-line immune defense within the CNS, these cells are involved in several mechanisms of neuronal homeostasis such as neural plasticity, synaptic remodeling and architecture, neurogenesis, and apoptosis (Paolicelli et al., 2011; Tay et al., 2018). As a consequence, their abnormal activation may impact key processes contributing to the pathophysiology of several diseases. In our study, the mRNA levels of CD11b, a marker of microglia activation, were increased after LPS in the hippocampus of male mice of both genotypes, with an expression profile comparable to that observed for the inflammatory cytokines IL-1β and TNF-α. Interestingly, only heterozygous female mice showed increased hippocampal levels of CD11b, a result that enlightens the enhanced susceptibility of mutant females. To confirm this modulation, we also analyzed the expression of fractalkine (CX3CL1) and its receptor (CX3CR1). CX3CL1 is mainly produced by neurons to bind its receptor on microglia surface, in order to control its activation state (Biber et al., 2007; Paolicelli et al., 2014). Interestingly, while fractalkine did not show clear modulations, CX3CR1 followed the expression profile of CD11b, in light of the specific expression of fractalkine receptor in this cellular population (Wolf et al., 2013). Accordingly, a different activation of microglia may contribute to the sex-dependent effect of LPS. Microglia cells show sex differences from the developmental stages in the rodent fetal brain: while males have more microglia in the developing brain, females boost the number of activated microglia during adulthood (Lenz and McCarthy, 2015). Moreover, adult microglia present several differences between males and females in terms of morphology, function, and transcriptional signature (Bilbo, 2017; Hanamsagar et al., 2017; Guneykaya et al., 2018). Our findings provide new information showing that BDNF may differently influence the microglia response in males and females, supporting the capability of the neurotrophin to act also at the immune level. In this sense, Lai et al. (2018) recently demonstrated that BDNF participates in the modulation of inflammatory homeostasis, with an anti-inflammatory activity on microglia through the erythropoietin (EPO) and sonic hedgehog (Shh) signaling pathways. Moreover, BDNF is also able to modulate the internal Ca^2+^ influx in microglia cells, thus controlling the release of pro-inflammatory molecules from activated microglia. These functions would suggest that BDNF might have—in the female—an anti-inflammatory impact through the control of microglial activation (Mizoguchi et al., 2009). Accordingly, the molecular susceptibility of heterozygous females to the LPS administration might be a potential consequence of differences in BDNF activity among the sexes. Interestingly, it has been shown that basal BDNF protein levels are doubled in females compared to males (Chourbaji et al., 2012), a finding that could be associated to the “masculinized” inflammatory response of female mutant mice lacking the neurotrophin. This observation supports the idea of a sex-dependent mechanism of action of the neurotrophin (Chan and Ye, 2017). In this sense, sex hormones might play a pivotal role in the control of BDNF expression. First, given that the BDNF gene contains an estrogen responsive element, the higher levels of circulating estrogens in females may control its expression with a transcriptional mechanism (Sohrabji et al., 1995). A caveat of our study is that we did not examine the estrous cycle of the female mice when LPS was administered. Further studies will be necessary to investigate the interaction between estrogens and BDNF in an inflammatory context, specifically taking into consideration the reported protective anti-inflammatory activity of these hormones (Vegeto et al., 2008; Villa et al., 2015). On the other hand, testosterone—whose levels are higher in males—can be converted into estrogen in specific brain regions by the enzyme aromatase, thus modulating BDNF expression with an estrogen-mediated mechanism also in males (Wei et al., 2017). Therefore, considering the sexual dimorphism of estrogen receptor and aromatase (Wu et al., 2009), BDNF transcription might be controlled with a sex-specific mechanism. Interestingly, not only the expression *per se*, but also the activation of the BDNF signaling differs from males to females. Specifically, Hill and co-workers demonstrated that male *BDNF*^+/–^ mice have increased activation of the cognate receptor of the neurotrophin, tropomyosin receptor kinase B (TrkB), when compared to female mutant mice. Despite the fact that the mechanism responsible for this increase has not been clarified, the authors suggest a potential contribution of steroid hormones (Hill and van den Buuse, 2011). This observation could, at least in part, explain the increased susceptibility in female mice in our study, since a basal increased TrkB activity in male mice could compensate for the lack of BDNF due to the mutation.

Based on the involvement of the immune/inflammatory system in the different vulnerability of the male and female brain to develop neurological and psychiatric disorders (Zagni et al., 2016), our results reveal a sex-dependent activity of BDNF on neuroinflammation suggestive of a potential new role for the neurotrophin in the sexual dimorphism of the central nervous system. Although the underlying molecular mechanism is still unknown, this finding might be of great interest for future studies aimed at developing therapeutic strategies for brain disorders with higher prevalence in women, in which BDNF plays a key role.

## Data Availability

No datasets were generated or analyzed for this study.

## Author Contributions

AR, PG, MR, and RM designed the study and wrote the protocol. AM and AT performed the LPS treatments; the behavioral test and the sample preparation; and carried out the related statistical analyses. AR, MP, and AT performed the gene expression assessments and carried out the related statistical analyses. AR, RM, PG, and MR wrote or contributed to the writing of the manuscript. All authors contributed to and have approved the final version of the manuscript.

## Conflict of Interest Statement

MR has received compensation as a speaker/consultant from the Lundbeck, Otzuka, Sumitomo Dainippon Pharma, and Sunovion, and he has received research grants from the Lundbeck, Sumitomo Dainippon Pharma, and Sunovion. The remaining authors declare that the research was conducted in the absence of any commercial or financial relationships that could be construed as a potential conflict of interest.
